# Skin Manifestations Among Individuals With Hepatitis C Infection

**DOI:** 10.7759/cureus.82902

**Published:** 2025-04-24

**Authors:** Erjola Toska, Cameron Minars, Suzanne I Riskin

**Affiliations:** 1 Department of Foundational Sciences, Nova Southeastern University Dr. Kiran C. Patel College of Osteopathic Medicine, Clearwater, USA

**Keywords:** cryoglobulinemia vasculitis, cutaneous, extrahepatic symptoms, hcv, hepatitis c, lichen planus, mixed cryoglobulinemia, porphyria cutanea tarda, pruritus, skin

## Abstract

Hepatitis C, an infection of the liver caused by the hepatitis C virus (HCV), is a growing global health concern, with an increasing annual incidence of primary disease and a notable likelihood of developing extrahepatic manifestations, including dermatological conditions. Studies have explored the relationship between hepatitis C infection and dermatologic manifestations, but there has been a limited number of comprehensive review manuscripts. This review aims to provide a comprehensive summary of studies conducted over a 10-year period, highlighting the relationship between hepatitis C infection and associated skin disorders. For this literature review, four databases, PubMed (NLM), EMBASE, CINAHL, and Web of Science (Core Collection), were searched for peer-reviewed articles written in English, involving human subjects, and published between 2013 and 2023. The search terms used were “(hepatitis C” OR “HCV”) AND (“skin” OR “cutaneous” OR “lichen planus” OR “cryoglobulinemia vasculitis” OR “porphyria cutanea tarda” OR “pruritus” OR “spider angiomas”)”. After removing duplicate articles from the four databases, the remaining articles were assessed for inclusion eligibility. A summary of findings revealed that individuals with hepatitis C exhibit a higher overall incidence of skin infections and experience these infections with increased severity in comparison to individuals without hepatitis C. Patients with hepatitis C face an increased risk of developing conditions such as psoriasis, onychomycosis, cutaneous lupus erythematosus, and lichen planus (LP). In addition, hepatitis C has been shown to be variably associated with oral lichen planus (OLP), suggesting the possibility of genotypic variations in HCV. Furthermore, hepatitis C has been identified as an etiological factor for cryoglobulinemia vasculitis, which manifests with skin symptomatology. These manifestations include palpable purpura, petechiae, vesicles, nodules, and livedo reticularis, predominantly affecting the lower extremities. This comprehensive review aims to provide insight into the association between hepatitis C infection and dermatological manifestations and to explore the presence and implications of skin conditions associated with the infection.

## Introduction and background

Hepatitis is a viral disease resulting from infection with one or more of the five recognized hepatitis viruses, including hepatitis A (HAV), hepatitis B (HBV), hepatitis C (HCV), hepatitis D (HDV), and hepatitis E (HEV). In the United States (U.S.), most viral hepatitis infections are due to HAV, HBV, and HCV. While hepatitis can present with acute symptoms such as nausea, malaise, abdominal pain, and jaundice, many cases remain asymptomatic, posing significant detection challenges [1]. Many individuals with hepatitis are unaware of their infection until they have already developed cirrhosis, progressed to end-stage liver disease, or developed hepatocellular carcinoma [1]. In addition, HCV and HBV can both lead to chronic infection.

HCV, which is a positive-sense single-stranded RNA virus, has been shown to result in 3 to 4 million new hepatitis C infections annually, making it a major global health problem [2]. The prevalence of chronic hepatitis C (CHC) is estimated at 2.9 to 3.9 million individuals in the United States alone [3]. HCV carries a high incidence of extrahepatic manifestations, which have been shown to occur commonly. The likelihood of developing at least one extrahepatic manifestation ranges from 40% to 74% among patients with hepatitis C infection over the course of the illness [4]. Involvement of the kidneys, skin, thyroid, eyes, joints, and nervous system has all been reported [4]. The pathology of these extrahepatic presentations remains an area of active research. It is hypothesized that both the host’s immune system response and cytopathic effects of the virus contribute to these manifestations [5]. Specifically, the underlying mechanisms associated with dermatologic manifestations are believed to involve an immune reaction to HCV. This can result in dermatological lesions linked to a variety of cutaneous conditions, which may differ between the acute and chronic phases of infection [2,5,6]. HCV is capable of evading the immune system, which ultimately results in chronic infection (CHC) [4]. Without treatment, the disease can progress to fibrosis, cirrhosis, hepatocellular carcinoma, liver failure, and death [3].

HCV has been broadly associated with various dermatological disorders such as lichen planus (LP), oral lichen planus (OLP), porphyria cutanea tarda (PCT), and mixed cryoglobulinemia (MC) [2,4,7]. Many individuals with hepatitis C infection exhibit no hepatic symptoms. As such, extrahepatic manifestations may be the first indication of HCV infection [4]. For example, the skin is involved in 95% of MC cases, typically manifesting as cutaneous vasculitis. This can present clinically as palpable purpura, petechiae in the lower extremities, or necrotic ulcerations [4].

Skin manifestations, as noted above, can often serve as an early indicator in the detection of systemic diseases, particularly hepatitis C infection. These dermatologic manifestations can encompass a wide range of conditions, including rashes, psoriasis, LP, and urticaria. While they may appear superficial, these skin conditions often reflect the underlying pathophysiology of disease and can negatively impact patient outcomes.

Using the National Health Insurance Research Database in Taiwan, researchers identified that HCV-infected patients had a 6.34-fold (95% CI, 5.30-7.58) increased risk for chronic inflammatory skin diseases compared to a control group, after adjusting for confounding variables [8]. The investigators assessed chronic inflammatory skin diseases (CISD) and concluded that HCV-positive patients had a significantly increased risk of developing LP (aHR = 13.14; 95% CI, 7.10-24.31), psoriasis (aHR = 6.42; 95% CI, 4.94-8.32), alopecia areata (aHR = 6.69; 95% CI, 4.28-10.44), and cutaneous lupus erythematosus (aHR = 13.48; 95% CI, 4.85-37.43) [8].

This is one of several studies that have identified varying associations between HCV infection and skin manifestations. As such, an unmet need exists to examine the broader body of evidence to better understand the relationship between HCV infection and dermatologic presentation. By evaluating and synthesizing the currently available literature, we aim to gain deeper insight into this link and its clinical implications. Understanding the underlying mechanisms of disease activity may enhance patient management and improve outcomes. Therefore, the purpose of this review is to address this unmet need, provide an overview of the current state of knowledge, and highlight potential directions for future research.

## Review

Methods

**Figure 1:**
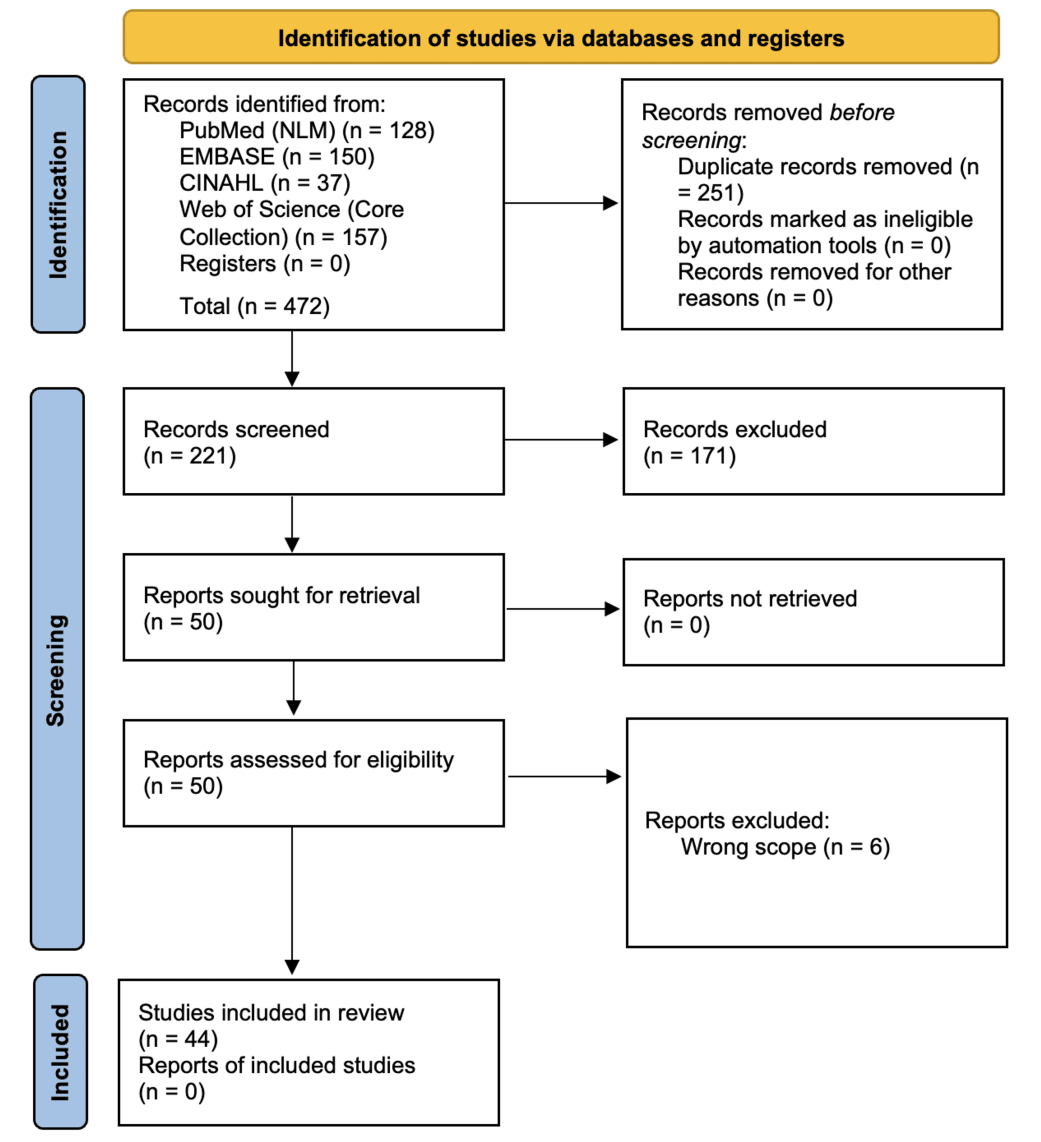
Preferred Reporting Items for Systematic Reviews and Meta-Analyses (PRISMA) diagram

Four databases, PubMed (NLM), EMBASE, CINAHL, and Web of Science (Core Collection), were searched for full peer-reviewed articles written in English about human subjects and published between January 2013 and December 2023. The initial approach was to search for terms in the title of articles: (“hepatitis C” OR “HCV”) AND (“skin” OR “cutaneous” OR “lichen planus” OR “cryoglobulinemia vasculitis” OR "porphyria cutanea tarda" OR “pruritus” OR “spider angiomas”). After assessing for duplicate articles from the four databases, the remaining articles were assessed for inclusion eligibility. Studies were excluded if they were written in non-English languages, were animal studies, abstracts/poster presentations, review articles, commentaries, editorials, or not centered around HCV cutaneous manifestations. The remaining articles were evaluated for this literature review. The process of article inclusion is depicted in the Preferred Reporting Items for Systematic Reviews and Meta-Analyses (PRISMA) diagram in Figure 1.

Review

Hepatitis C Virus and General Skin Manifestations

HCV infections may present with a range of skin manifestations. A retrospective analysis of 3,496 subjects undergoing HCV antibody surveillance identified 150 subjects with skin diseases [9]. Among these 150 subjects, 6.7% were positive for anti-HCV antibodies, indicating a significant association (p = 0.0053) between types of skin diseases and HCV infection in patients with skin diseases [9]. In addition, a prospective study evaluated 95 HCV-positive patients with diabetes and 68 HCV-negative patients with diabetes for skin infections, specifically excluding those undergoing interferon treatment or presenting with other systemic diseases [10]. Over a three-year period, patients were examined for any cutaneous infection. The investigators identified a higher incidence of skin infections among the HCV-positive patients. Specifically, HCV-positive patients demonstrated a 14.6% increase in viral infections and an 8.7% increase in fungal skin infections [10]. Overall, this study indicated that HCV-positive patients with diabetes had a significant increase in cutaneous infections compared to their HCV-negative counterparts with diabetes. In addition, these infections presented with increased severity and were associated with a higher resistance to treatment, requiring more aggressive treatment courses [10]. For example, the study noted that fungal infections, such as onychomycosis or tinea corporis, in HCV-positive patients with diabetes showed marked resistance to itraconazole and topical antifungal medications, requiring systemic therapy such as terbinafine. Both studies demonstrate that individuals who are HCV-positive have a higher risk of skin manifestations, which may be resistant to treatment and require more intensive treatment regimens.

Studies conducted in countries with a high prevalence of HCV infection have also demonstrated a link between HCV and skin disorders. Pakistan has one of the highest populations of individuals with hepatitis C, with more than 11% of the population infected with HCV [11]. In a cross-sectional study conducted in Pakistan, researchers followed 100 diagnosed hepatitis C patients for six months. The investigators found that cutaneous manifestations were frequent among HCV patients, with many presenting with multiple skin conditions, including generalized pruritus, LP, urticaria, and porphyria cutanea tarda (PCT) [12]. Another study conducted in Pakistan supported this observation, in which the researcher found that HCV patients had higher odds of having skin diseases compared with controls, displaying an odds ratio of 4.85 (CI 1.95-12.12). This indicated a notable prevalence of skin diseases among HCV-infected individuals [13]. In a cross-sectional study conducted in 2019 in Pakistan involving 212 participants, the researchers investigated the frequency of cutaneous manifestations among individuals with hepatitis C. The investigators found that 87.3% of patients with HCV infection had cutaneous manifestations [11]. Pruritus was the most common cutaneous manifestation, with a frequency of 33.96%, followed by LP, with a frequency of 23.5% [13]. These three studies conducted in Pakistan highlight the link between hepatitis C infection and dermatologic manifestations.

Research in Egypt, which also presents with a high prevalence of HCV infection (14.7%), similarly reports a significant association with dermatologic manifestations, with 20% of the HCV-positive patients developing cutaneous manifestations [14]. Specifically, one study conducted in Egypt that included 1,000 HCV-positive patients found that 36.9% presented with cutaneous manifestations. Pruritus was the most common manifestation, followed by generalized hyperpigmentation (27.6%) and psoriasis (5.4%) [15]. The combination of studies conducted in Pakistan and Egypt highlights a link between HCV infections and multiple cutaneous manifestations, which warrants further investigation as well as consideration in the management of patients with HCV infection and/or skin manifestations.

Lichen Planus and Oral Lichen Planus

LP is characterized by chronic inflammation and is likely considered an autoimmune mucocutaneous condition. OLP, which is characterized by chronic inflammation and affects the oral cavity, has been demonstrated to be associated with systemic conditions such as diabetes, hypertension, systemic lupus erythematosus, and hepatitis [5,16]. An association between HCV infection and OLP has been suggested. Several studies have questioned the strength of the relationship between autoimmune LP and HCV [17]. Within the broader context of HCV and the host immune response, T-regulatory lymphocytes play a pivotal role in determining both extrahepatic manifestations, such as immune-mediated skin conditions, and the overall progression of HCV infection [18]. A study examining the immune system response in chronic hepatitis C (CHC) and its relationship to immune-mediated cutaneous manifestations specifically focused on the frequency of CD4^+^CD25 high FOXP3^+^ T-regulatory lymphocytes [18]. Among the CHC patients studied, including 30 patients with skin manifestations and 28 patients without, there was a significantly lower frequency of Treg cells in CHC patients with skin manifestations compared to those without, indicating a higher prevalence of chronic hepatic insult among patients exhibiting skin manifestations [18].

Another study comparing the severity of atrophic/erosive lesions in OLP-HCV patients with idiopathic OLP patients showed that the OLP-HCV group exhibited a higher relationship between the counts of CD8/FOXP3⁺ T cells per mm² (p = 0.018) and counts of CD8⁺ T cells per mm² (p = 0.034) [19]. The relationship indicates a higher number of CD8⁺ T cells in OLP-HCV patients, but lower FOXP3⁺ expression [19]. These findings identify the role of the host immune response in shaping the clinical manifestations and severity of HCV-related immune-mediated skin conditions, offering valuable insights to further understand and manage these complex interactions in affected individuals.

LP can affect various areas, including the skin, oral mucosa, scalp, and nails [5], displaying diverse clinical presentations such as pruritus and purple, polygonal-shaped, planar papules or plaques [5,20]. The lesions present in LP are thought to consist of inflammatory infiltrate and are a source of excess reactive oxygen species (ROS) production [21]. ROS can be generated by epidermal keratinocytes, leading to a potential link with HCV infection [21]. An observational study investigated the pathogenesis of oxidative stress in LP and the associations between LP and HCV infection using serum levels of 4-hydroxynonenal (4-HNE) and symmetric dimethylarginine (SDMA) as markers of oxidative stress [21]. The researchers found that HCV amplified oxidative processes in LP, creating an imbalance between oxidants and antioxidants. The oxidative imbalance likely impacted the pathogenesis of LP [21].

In another study evaluating the cellular proliferative potential of OLP lesions in association with HCV, four groups were studied: HCV-positive patients with no presence of OLP lesions and no current treatment; HCV-positive patients under treatment with interferon and ribavirin; HCV-negative patients with reticular OLP lesions; and a control group of HCV-negative patients with no clinical signs of OLP [22]. The results suggested that previously treated HCV-positive patients demonstrated higher cellular proliferative activity, indicating a possible association between HCV and histopathological manifestations [22]. The study indicated that interferon and/or ribavirin treatment for HCV may contribute to the clinical presentation of OLP lesions through an increase in the number of nucleolar organizer regions (chromosomal regions active during interphase within the nucleolus in oral mucosa cells), possibly linking OLP with anti-HCV treatment [22].

These findings contribute to the understanding of the complex connections between LP, oxidative stress, HCV infection, and LP treatments, offering insights into the pathogenesis behind skin manifestations in patients with hepatitis C infection.

Cryoglobulinemia Vasculitis

Mixed cryoglobulinemia (MC) is a systemic vasculitis characterized by the deposition of circulating immune complexes in small and medium-sized blood vessels and an overproduction of IgM with rheumatoid activity of B cells [4,23]. MC can result in inflammation of blood vessels, which is known as MC vasculitis, and is common among patients with hepatitis C infection [23]. Still, the underlying mechanisms of the disease activity of MC have yet to be fully elucidated, and its links to HCV remain unclear. Circulating mixed cryoglobulins have been shown to be present among 40% to 60% of patients with HCV infection, with cryoglobulinemia vasculitis only observed in 5% to 10% of cases [24]. MC clinically presents as “MC syndrome,” which refers to the presence of purpura, arthralgia, and asthenia, and possible serious renal and neurological involvements [23].

It has been suggested that the presence of MC among patients with chronic HCV infection can be associated with cutaneous vasculitis [6]. Meanwhile, the exact prevalence of MC-induced cutaneous manifestations remains unclear. It has been suggested that MC can present as palpable purpura, leukocytoclastic vasculitis, or petechiae in the lower extremities [6,24]. In one study, this prevalence was investigated by evaluating the blood serum of 118 participants with HCV who were exhibiting cutaneous manifestations. The researchers found that 10.169% of these patients were cryopositive, a positive test result for cryoglobulins in the blood, suggesting a lower prevalence of MC compared to previous studies [6]. Vasculitic lesions in the lower legs, such as palpable purpura, vesicles, nodules, and livedo reticularis, were found to be significantly more prevalent among patients with MC, which is consistent with the previous study connecting hepatitis C and cryoglobulinemia vasculitis [6]. In another study of 148 HCV-cryoglobulinemia vasculitis patients, the cutaneous clinical features present included purpura (57.4%) and skin necrosis (10.1%), underlining the findings of previous research [25]. In a smaller study of 27 patients with HCV-CV, the clinical features found were purpura (89%) and skin ulcers (15%) [24]. The studies suggest significant dermatological presentations in HCV-infected individuals with the presence of MC, which warrants further research.

Suggested treatments for MC associated with HCV infection include directly targeting HCV using direct-acting antivirals (DAA) or targeting the underlying B-cell autoimmune activity with rituximab [26]. DAA aims to decrease immune complex formation among individuals with HCV, which can also result in a reduction in symptoms associated with cutaneous vasculitis [23]. This aligns with findings from another study that showed targeting HCV led to a decrease in vasculitic manifestations, suggesting that immunosuppressive therapy may only be necessary in more severe cases [27]. However, it is important to note that during the course of treatment, subsequent cutaneous manifestations may also occur as adverse events of medications used in the treatment of viral hepatitis, which could be linked to abnormal liver function [7].

Pruritus

Pruritus is a sensation that induces the need to rub or scratch the skin, which is conducted via specific afferent fibers in the central nervous system [28]. A study evaluated 110 individuals infected with HBV, HCV, or both to assess the clinical manifestations of pruritus and chronic viral hepatitis [28]. Among the 110 subjects, 20% reported experiencing pruritus, and among that subset, 59.1% had hepatitis C [28]. The study noted that the majority of subjects with pruritus had secondary skin changes due to scratching [28]. This suggests a potential link between chronic hepatitis C and pruritus as the initial skin disorder. Supporting this finding, another study observed that patients with chronic hepatitis C significantly experienced nocturnal pruritus (58.4% compared to 5.5% in the control group of non-hepatitis patients) with a p-value < 0.0001, emphasizing a strong association between hepatitis C and pruritus [29]. Subsequent studies conducted in Pakistan aimed to determine the frequency of HCV seropositivity in patients with pruritus, revealing that 23% of patients experiencing pruritus tested positive for HCV [30]. An observational study investigating uremic pruritus also found a significant association between HCV infection and severe uremic pruritus [31]. These collective findings highlight the potential connection between HCV infection and the prevalence of pruritus, suggesting a noteworthy association between hepatitis C and the manifestation of pruritus, contributing to a deeper understanding of the dermatological impact of viral infections.

Porphyria Cutanea Tarda

Porphyria Cutanea Tarda (PCT) is a skin condition associated with painful blisters on the skin due to exposure to sunlight. PCT results from decreased activity of the uroporphyrinogen decarboxylase (UROD) enzyme, leading to a buildup of uroporphyrinogen [5,32]. Decreases in UROD enzyme activity are thought to be attributable to the presence of oxidative stress and mild to moderate elevation of iron in the liver, resulting in the production of an inhibitor [5,32]. HCV infection has been demonstrated to increase oxidative stress in hepatocytes and suppress hepcidin production. This increases iron absorption and has been observed in 50%-70% of PCT cases in the United States [32]. Research evidence suggests that HCV often precedes the clinical manifestations of PCT, indicating a potential role of HCV in disrupting porphyrin metabolism or chronically impacting the cytochrome P450 system [5]. This supports the presence of subclinical porphyrinuria among individuals infected with HCV and highlights a potential mechanism for skin manifestations in patients with hepatitis C infection [33].

Onychomycosis

Onychomycosis is defined as a fungal infection of the nail and causes discoloration and/or thickening of the nail plate [34]. The clinical manifestations of onychomycosis have been shown to be more prevalent among patients with HCV (17.8%) as compared with individuals who are HCV-negative [10]. The researchers treated individuals with and without HCV infection for onychomycosis with itraconazole and found that more HCV-positive patients presented with poor or mild improvement (79.2%) as compared with the HCV-negative group, in which 20% of patients demonstrated mild improvement [10].

Regional Hepatitis C Virus Genome

The relationship between hepatitis C and skin manifestations, including LP and OLP, across multiple studies from different regions of the world has demonstrated varying outcomes. Studies conducted in Spain, Brazil, Thailand, and Japan identified a positive association between hepatitis C and skin manifestations, including LP and OLP [16,35]. Research conducted in Pakistan identified that HCV may play an etiological role in the development of LP [36]. Another study conducted in Brazil reported a higher percentage of positive HCV serology among patients with LP [37]. Notably, a cross-sectional study in Pakistan revealed that 14% of patients with LP were seropositive for HCV [20], while a separate study showed that there was a high frequency of positive HCV in LP patients [38]. An investigation comparing the frequency between HCV and OLP, a sample of 237 OLP patients and age/gender-matched 948 control individuals (non-OLP patients) were screened over a three-year period for HCV. The researchers found a significantly higher frequency of HCV among OLP patients as compared to controls (frequency of 19.8% vs. 2.1%) [39]. Conversely, other regions, including the Netherlands [16], the United States [16], Poland [40], China [41], central Germany [17], and southeast Iran [42] have identified a negative association between HCV infection and skin manifestations, including LP and OLP. In addition, studies conducted within the northeast region of Iran and a longitudinal clinical study conducted in Brazil found no significant relationship between hepatitis C infection and OLP [2,43]. The divergent findings across different geographic regions indicate the possibilities of a genotypic variation of the infecting HCV, which may also play a role in the variability of the association of OLP [16]. The study performed in Germany noted that the results they had could have been due to the small population size [17]. Articles that support the association of HCV and LP recommend that patients with LP should get screened for an early diagnosis of HCV along with ALT and AST labs [44].

Through diverse investigations into the connection between HCV and LP, a genome-wide association (GWAS) study and gene polymorphisms study in Japan and Egypt, respectively, have attempted to identify genetic variants associated with HCV-related LP [14,45]. With a sample size of 261 patients in Japan, a statistical significance with a p < 0.0001 found that single-nucleotide polymorphisms rs884000 in the NRP2 locus and rs538399 in the IGFBP4 locus were linked with the risk of HCV-associated LP [45]. The association of HLA-DR and DQ genes with HCV-positive OLP was also found. In an Egyptian study with a total of 60 patients, HCV seropositive patients had significantly higher OLP activity [14]. The study examined Interleukin 8 (IL-8) gene polymorphism rs4073 and suggested that the hepatitis C virus infection increases IL-8 production during the host’s immune response, thereby exacerbating the disease in the manifestation of erosive oral lichen planus [14]. Further establishing the research of morphological variants of lichen planus in 200 HCV-positive patients, among the isolated variants, hypertrophic LP was the largest clinical type with 25% frequency, followed by classic at 21% and oral at 17.5% [46]. Fewer present LP in isolated variants were pigmented at 11% frequency and atrophic at 4% [46]. These studies offer potential explanations for the contentious discourse surrounding the association between HCV and OLP across different geographical regions, showcasing that distinct HCV variants with diverse gene polymorphisms could contribute to the risk of OLP in HCV-infected individuals.

## Conclusions

This review compiled and summarized existing studies and research focused on the relationship between hepatitis C infection and skin manifestations. Findings of this review highlighted the association of both acute and chronic hepatitis C infection with multiple dermatological conditions, including psoriasis, onychomycosis, cutaneous lupus erythematosus, and LP. In addition, hepatitis C has been shown to be associated with cryoglobulinemia vasculitis, which commonly manifests on the lower extremities as palpable purpura, petechiae, vesicles, nodules, and livedo reticularis. The association between hepatitis C and OLP varied across patients, which warrants future research efforts. Specifically, suggested future studies could explore whether a genetic or genotypic variation exists within individuals with hepatitis C, or within the HCV itself, in relation to OLP. Overall, skin manifestations may serve as an indication of chronic hepatitis C infection and should be further evaluated as potential diagnostic markers of underlying hepatitis C infection.
